# Electroconvulsive Therapy (ECT) and Repetitive Transcranial Magnetic Stimulation (rTMS), Benefits and Adverse Effects in Patients with Depression: A Scoping Review

**DOI:** 10.3390/jcm15135194

**Published:** 2026-07-02

**Authors:** Miguel Esteban Carrera-Aguilar, Erick Castro, Diana Álvarez-Mejía, Roberto Martín Vargas-Villacís, Martina Coronel, Marcelo Pinto-Proaño, José Arcentales, Jose E. Leon-Rojas

**Affiliations:** 1 NeurALL-Research Group, Quito 170157, Ecuador; micarreraag@uide.edu.ec (M.E.C.-A.); rovargasvi@uide.edu.ec (R.M.V.-V.); marticoronelh@gmail.com (M.C.);; 2 Escuela de Medicina, Universidad Internacional del Ecuador, Jorge Fernández, Quito 170411, Ecuador; 3 Instituto de Neurobiología, Universidad Autónoma de México, Campus UNAM 3001, Juriquilla, Querétaro 76230, Mexico; 4 Facultad de Psicología, Universidad de Salamanca, Patio de Escuelas, 1, 37008 Salamanca, Spain; 5 Escuela de Medicina, Universidad San Francisco de Quito, Campus Cumbayá, Diego de Robles, Quito 170901, Ecuador; 6 Grupo de Investigación Bienestar, Salud y Sociedad, Escuela de Psicología y Educación, Universidad de las Américas, Quito 170124, Ecuador

**Keywords:** major depressive disorder, treatment-resistant depression, electroconvulsive therapy, repetitive transcranial magnetic stimulation, neuromodulation, depression severity, adverse effects, precision psychiatry, brain stimulation, non-pharmacological treatment

## Abstract

**Background:** Major depressive disorder, particularly in its treatment-resistant form, remains a leading cause of global disability. When pharmacotherapy and psychotherapy fail, neuromodulation techniques such as electroconvulsive therapy (ECT) and repetitive transcranial magnetic stimulation (rTMS) are increasingly utilized. However, variability in protocols and outcome reporting continues to generate uncertainty regarding their comparative benefits and safety profiles. **Objective:** To comprehensively map and synthesize the available evidence on the clinical benefits and adverse effects of ECT and rTMS in adults with major depressive disorder and treatment-resistant depression. **Methods:** A scoping review was conducted following PRISMA-Sc guidelines and registered in PROSPERO; PubMed–MEDLINE, Scopus, and the Virtual Health Library were searched from inception to October 2022. Observational and experimental studies evaluating ECT and or rTMS in adults with depressive disorders were included. Data were extracted on study design, population characteristics, stimulation parameters, clinical outcomes, and adverse effects. Methodological quality was assessed using National Heart, Lung, and Blood Institute tools. **Results:** A total of 165 studies comprising 10,701 participants were included. ECT and rTMS were consistently associated with clinically meaningful reductions in depressive symptom severity across heterogeneous protocols. ECT demonstrated the most robust response rates, particularly in treatment-resistant and severe depression, while rTMS showed substantial efficacy with a more favorable safety profile. Adverse effects were more frequent and severe with ECT, including transient cognitive disturbances and cardiovascular complications, whereas rTMS was predominantly associated with mild, self-limited side effects such as headache and scalp discomfort. Considerable heterogeneity in stimulation parameters and diagnostic subgroups was observed across studies. **Conclusions:** Both ECT and rTMS represent effective neuromodulation strategies for major depressive disorder and treatment-resistant depression. ECT remains the most potent intervention in highly refractory cases, whereas rTMS offers a less invasive alternative with strong tolerability. Standardization of stimulation protocols, biomarker-informed stratification, coadjuvancy analysis, and long-term controlled studies are necessary to refine clinical positioning and advance precision neuromodulation in depression care.

## 1. Introduction

Major depressive disorder (MDD) is a common and disabling psychiatric disorder characterized by persistent depressed mood, anhedonia, cognitive and vegetative symptoms, functional impairment, and an increased risk of recurrence and suicide [1]. Although many patients improve with antidepressant pharmacotherapy, psychotherapy, or their combination, a substantial proportion do not achieve adequate symptomatic remission. Treatment-resistant depression (TRD) is commonly defined as an insufficient clinical response to at least two antidepressant regimens administered at adequate dose and duration, and it represents one of the most challenging scenarios in contemporary mental health care [2]. In this context, non-pharmacological interventions have gained clinical relevance, particularly for patients with severe, recurrent, psychotic, suicidal, or pharmacoresistant depressive episodes.

Electroconvulsive therapy (ECT) and repetitive transcranial magnetic stimulation (rTMS) are two of the most widely used neuromodulatory interventions for depressive disorders [3,4,5,6,7]. ECT remains one of the most effective treatments for severe and treatment-resistant depression, especially when rapid clinical response is required or when multiple pharmacological strategies have failed [4,7]. By contrast, rTMS is a non-invasive brain stimulation technique that modulates cortical excitability through repeated magnetic pulses, most commonly targeting prefrontal networks implicated in mood regulation [3,5]. Although TMS has also been investigated across several psychiatric and neurological conditions, including schizophrenia, anxiety disorders, pain, Parkinson’s disease, stroke, multiple sclerosis, and epilepsy, its application in depression has become one of its most clinically relevant and evidence-supported uses [5]. In depressive disorders, rTMS protocols are usually classified according to stimulation frequency, cortical target, laterality, coil type, stimulation intensity, and treatment schedule, with high-frequency left dorsolateral prefrontal cortex stimulation and low-frequency right dorsolateral prefrontal cortex stimulation representing the most established approaches [3,5,6].

Despite the increasing clinical use of both ECT and rTMS, direct interpretation of the literature remains challenging because studies vary substantially in patient populations, depressive subtypes, treatment resistance definitions, stimulation parameters, outcome measures, follow-up duration, and adverse-effect reporting. These sources of heterogeneity limit the suitability of a purely comparative meta-analytic approach and support the use of a scoping review to map the breadth of available evidence, identify recurring protocols, and clarify safety and outcome reporting patterns. Therefore, this scoping review aimed to map the range of ECT and rTMS protocols, reported clinical outcomes, and adverse effects in adults with depressive disorders, with special attention to treatment-resistant depression.

## 2. Methods

Our scoping review was conducted according to PRISMA-ScR guidelines [8] and registered in PROSPERO (CRD42023470774). A completed PRISMA-ScR checklist is provided in Supplementary Material Table S3. The review was designed to map the breadth of available evidence on ECT and rTMS in adult depressive disorders, rather than to generate pooled estimates of comparative efficacy. Accordingly, we focused on identifying the range of study designs, patient populations, stimulation protocols, clinical outcomes, and adverse effects reported in the literature. The methodological structure included definition of the review question, identification of relevant studies, selection of eligible evidence sources, extraction and charting of key data items, descriptive synthesis of findings, and presentation of clinically relevant evidence gaps.

### 2.1. Eligibility Criteria and Language

We included studies conducted in adult humans, aged 18 years or older, with depressive disorders in which depression was the primary clinical condition or the explicit target of ECT and/or rTMS treatment. Eligible diagnoses included major depressive disorder, treatment-resistant depression, unipolar depressive disorder, bipolar depression in the depressive phase, postpartum depression, vascular depression, late-life depression, refractory or medication-resistant depression, and depressive syndromes associated with neurological or medical comorbidities, provided that the diagnosis of depression was clearly stated and treatment outcomes were reported. Observational studies, including cross-sectional and cohort designs, and experimental studies, including randomized clinical trials, were eligible for inclusion. Articles in English or Spanish were included, with no restriction according to year of publication. We excluded literature reviews, scoping reviews, systematic reviews, letters to the editor, conference abstracts, case reports, and case series. Articles were also excluded when the diagnosis of depression was not clearly stated or validated, when the intervention was not ECT or TMS/rTMS, or when treatment outcomes or adverse effects were not reported with sufficient clarity.

For treatment-resistant depression, we accepted the definition used by each original study, including terms such as treatment-resistant, refractory, drug-resistant, or medication-resistant depression, when explicitly defined or clearly stated by the study authors. Because definitions of TRD varied across studies, especially across different publication years and study designs, we did not retrospectively apply a single uniform operational threshold to all included articles. This approach was consistent with the scoping objective of mapping the available evidence across heterogeneous clinical and methodological contexts.

### 2.2. Information Sources and Search Strategy

We queried three databases, PubMed–MEDLINE, Scopus, and the virtual health library (BVS), from inception until October 2022. Our research strategy included a mix of free-text and medical subheadings (MeSH) terms combined with parentheses and Boolean operators; search terms were derived from the following keywords: depression, electroconvulsive therapy, transcranial magnetic stimulation, treatment, and clinical effects (outcomes). The complete search strategy used to query each database can be found in the Supplementary Materials Table S1.

### 2.3. Data Management and Selection Process

Results of the literature search were imported to the online software Rayyan.ai to facilitate collaborative data analysis and mitigate user error. Using this software, four independent and blinded reviewers screened the titles, abstracts, and keywords against the eligibility criteria; any discrepancies were solved by a different reviewer and through mutual consensus. Afterwards, five independent and blinded reviewers assessed the filtered articles by reading the full text; any discrepancies were solved by discussion and mutual consensus.

### 2.4. Data Items and Synthesis

The following information was extracted from a Microsoft Excel spreadsheet (version 16.78.3): author, contact, title, DOI, year, country, type of study, time since diagnosis, type of depression, type of treatment (TMS or ECT) and modality, other associated treatments, benefits of treatment, adverse effects, and possible causes of depression. For each article, treatment-specific characteristics of both ECT and TMS were collected, and these data were grouped into tables for comparison with information on the benefits and adverse effects of each method. We also considered sociodemographic characteristics of the population studied and the methods by which the depressive symptoms were evaluated.

Outcome data were synthesized descriptively. When patient counts were reported, they were aggregated to map the frequency and direction of reported outcomes across intervention categories. These cumulative counts were not treated as pooled response rates, were not weighted by study design or methodological quality, and were not used to calculate comparative effect estimates. Because included studies varied substantially in populations, stimulation parameters, outcome scales, follow-up periods, and definitions of response or symptom improvement, the aggregated counts should be interpreted as descriptive evidence-mapping summaries rather than meta-analytic estimates.

### 2.5. Methodological Quality Appraisal

Methodological quality was appraised using the study quality assessment tools developed by the National Heart, Lung, and Blood Institute, NHLBI, available at https://www.nhlbi.nih.gov/health-topics/study-quality-assessment-tools, accessed on 10 September 2025 15 November 2025. The tool was selected according to the study design. Randomized and controlled intervention studies were assessed using the NHLBI Quality Assessment Tool for Controlled Intervention Studies, while observational cohort and cross-sectional studies were assessed using the NHLBI Quality Assessment Tool for Observational Cohort and Cross-Sectional Studies. No checklist items were modified or removed. All eligible studies were independently assessed by two reviewers, and discrepancies were resolved by discussion with a third reviewer. The appraisal was used to contextualize the methodological robustness of the mapped evidence and was not used as an exclusion criterion. Because this was a scoping review, the appraisal did not inform quantitative weighting or meta-analytic pooling.

For descriptive purposes, we summarized the proportion of affirmative responses across applicable NHLBI items for each study. Studies with 80% or more affirmative responses were described as having higher methodological quality, studies with 50% to 79% affirmative responses were described as having moderate methodological quality, and studies with less than 50% affirmative responses were described as having lower methodological quality. These categories were used as an internal descriptive framework and should not be interpreted as formal NHLBI risk-of-bias categories.

## 3. Results

### 3.1. Selection Process

**Figure 1:**
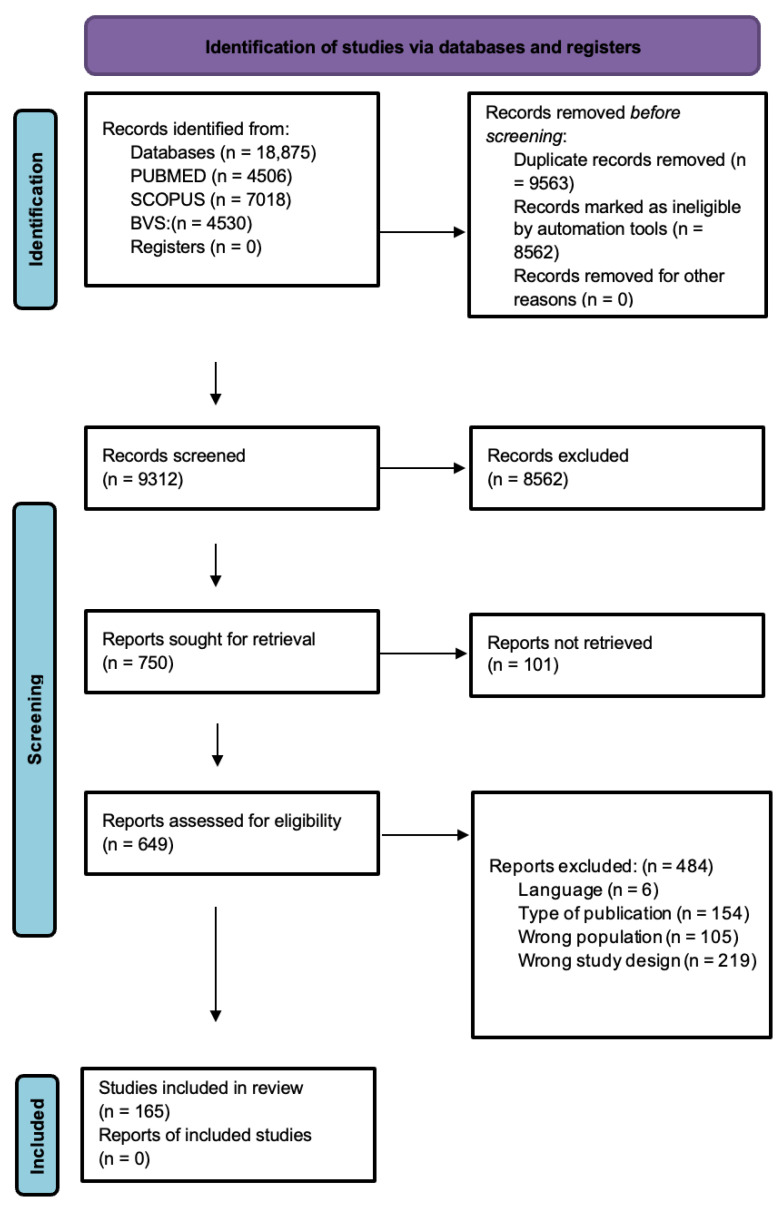
PRISMA 2020 Flow Diagram showcasing the results of the selection process.

PubMed, Scopus, and BVS identified a total of 18875 articles; from those, 9312 remained after conducting deduplication and were subjected to two screening processes (title/abstract and full-text), leaving 165 articles which varied in design and included a total of 10,701 participants [9,10,11,12,13,14,15,16,17,18,19,20,21,22,23,24,25,26,27,28,29,30,31,32,33,34,35,36,37,38,39,40,41,42,43,44,45,46,47,48,49,50,51,52,53,54,55,56,57,58,59,60,61,62,63,64,65,66,67,68,69,70,71,72,73,74,75,76,77,78,79,80,81,82,83,84,85,86,87,88,89,90,91,92,93,94,95,96,97,98,99,100,101,102,103,104,105,106,107,108,109,110,111,112,113,114,115,116,117,118,119,120,121,122,123,124,125,126,127,128,129,130,131,132,133,134,135,136,137,138,139,140,141,142,143,144,145,146,147,148,149,150,151,152,153,154,155,156,157,158,159,160,161,162,163,164,165,166,167,168,169,170,171,172,173,174]. The full screening process can be found in Figure 1.

### 3.2. Methodological Quality Appraisal

Methodological quality was appraised in all 165 included studies using the appropriate NHLBI tool according to study design. Using our predefined descriptive framework, 23% of studies were classified as having higher methodological quality, 75% as having moderate methodological quality, and 2% as having lower methodological quality. These categories were used to contextualize the narrative synthesis and should not be interpreted as formal NHLBI risk-of-bias categories (Supplementary Table S2).

### 3.3. Reporting Frequency of TMS and ECT in the Literature

The most reported therapy was TMS alone, with a total of 4862 patients, of which 2751 were females and 1887 males [9,10,11,12,13,14,15,16,17,18,19,20,21,22,23,24,25,26,27,28,29,30,31,32,33,34,35,36,37,38,39,40,41,42,43,44,45,46,47,48,49,50,51,52,53,54,55,56,57,58,59,60,61,62,63,64,65,66,67,68,69,70,71,72,73,74,75,76,77,78,79,80,81,82,83,84,85,86,87,88,89,90,91,92,93,94,95,96,97,98,99,100,101,102,103,104,105,106,107,108,109,110,111,112,113,114,115,116,117,118,119,120,121,122,123,124,125,126,127,128,129,130,131,132,133,134,135,136,137,138,139,140,141,142,143,144,145,146,147,148,149,150,151,152,153,154,155,156,157,158,159,160,161,162,163,164,165,166,167,168,169,170,171,172,173,174]. Overall, the female gender was predominant, with a 1.62 female:male ratio [3,5,9,10,11,13,14,15,16,17,18,19,20,21,22,23,25,26,32,34,36,37,38,39,40,41,42,43,44,47,48,49,50,51,52,53,54,55,57,58,59,61,62,64,65,66,67,68,69,70,71,72,73,75,76,77,78,79,80,81,82,83,84,86,87,88,89,90,92,93,94,95,96,97,98,99,100,101,102,103,104,105,107,108,109,110,111,113,114,115,116,117,118,119,120,121,122,123,124,125,126,128,129,130,131,132,133,134,135,137,138,139,140,141,143,144,145,146,147,148,149,150,151,152,153,154,155,156,157,158,159,160,162,163,165,166,167,168,169,170,172,173,175,176,177,178,179,180]. A total of 1090 patients were reported without a defined gender [6,24,27,28,29,30,31,33,35,45,46,56,63,74,85,91,106,112,127,136,142,164,171,174]. After TMS, ECT alone was the second most used therapy with 3987 patients in total, followed by coadjuvant pharmacological treatment in 1132 patients. Table 1 showcases the absolute frequency of articles wherein these types of therapies were reported.

Most of the analyzed population was diagnosed with major depressive disorder, accounting for 69.7% of participants, followed by treatment-resistant depression, which represented 21.8% of cases. The remaining proportion corresponded to clinical variants or subtypes derived from these two primary diagnostic categories, characterized by greater severity, chronicity, or resistance to conventional pharmacological treatment [6,12,135,152]. These findings are consistent with previous literature indicating that MDD constitutes the most frequently studied depressive disorder in neuromodulation research, while TRD represents a substantial subgroup due to its association with poorer outcomes and the need for advanced therapeutic interventions such as ECT and TMS [135,161,162].

### 3.4. Reported Benefits of ECT and TMS

#### 3.4.1. General Characteristics

A total of 81 different scales were included in our review; of these scales, most represented corresponds to the Hamilton scales (in 64.84% of included articles) [3,9,11,16,21,23,25,26,27,28,31,32,33,34,35,36,38,39,40,41,42,44,46,47,49,54,55,56,57,58,60,61,63,65,66,67,71,73,74,75,79,80,82,87,88,89,91,92,95,96,97,98,99,100,103,105,106,107,108,109,110,111,113,114,117,118,120,121,122,123,127,128,129,130,132,133,136,137,138,139,140,143,144,146,147,148,149,152,154,155,156,158,160,161,162,163,167,168,169,172,173,174,175,176,177,179,180]. The second most used scale was the 24 items Montgomery–Asberg Depression Rating Scale (MADRS) (in 14.54% of included articles) [13,14,15,19,29,30,37,45,51,55,68,69,77,90,94,97,99,104,123,131,144,168,169,171], followed by the 20 items Beck Depression Inventory (BDI) (in 12.12% of included articles) [20,21,23,28,29,35,37,65,83,96,103,108,110,112,117,125,130,133,144,155]. It should be noted that many articles used a varied set of different scales to evaluate participants, therefore the reported count of these scales do not reflect the superiority of certain scales over others.

The following counts are presented as descriptive evidence-mapping summaries. They indicate how many participants were reported as having symptom reduction, clinical improvement, or other beneficial outcomes across the included studies. These figures should not be interpreted as pooled response rates or direct comparative efficacy estimates, because the included studies used heterogeneous diagnostic criteria, stimulation protocols, outcome scales, follow-up durations, and definitions of response.

In general, we found that both ECT and TMS provide clinically relevant benefits for patients diagnosed with depressive disorders. Studies including exclusively active treatment samples reported that, across 55 articles evaluating ECT, a total of 2781 patients experienced a reduction in depressive symptoms, 60 demonstrated improvements in standardized depression rating scales, 437 reported enhancements in quality of life, 161 identified additional functional or psychosocial benefits, and 20 individuals showed increased levels of brain-derived neurotrophic factor (BDNF) [2,7,21,135,152]. In comparison, analyses restricted to active TMS treatment also revealed substantial clinical improvement; across 95 articles, 3748 patients experienced symptom reduction, 246 showed improvement in depression severity scores, 164 reported increased quality of life, and 48 identified other beneficial outcomes associated with treatment [3,6,34,35,36,37,38,39,40,41,103,153]. These findings reinforce the growing evidence supporting TMS as an effective neuromodulation strategy for depressive disorders.

In addition to efficacy outcomes, differences in adverse effect profiles between therapies were consistently reported. TMS was generally associated with mild, transient, and self-limited side effects, whereas ECT was linked to more severe adverse events, including cardiac and neurological complications [10,11,114,135]. This distinction is particularly relevant for clinical decision-making, especially in vulnerable populations such as older adults or patients with significant medical comorbidities, where treatment tolerability and safety considerations are paramount [12,161,165].

#### 3.4.2. Electroconvulsive Therapy (ECT) Alone

Within the ECT there are certain characteristics that contribute to the treatment and its results; for example, it is important to consider the area of stimulation such as the fronto-temporal, frontal, and temporal lobes or if the therapy is applied unilaterally or bilaterally. In certain cases, in the literature, such information was not readily available. The largest subgroup of articles reporting on the use of ECT consisted of patients receiving unspecified ECT modalities, in which 2058 participants (99.5%) experienced a reduction in depressive symptoms, while only 11 patients (0.5%) did not show improvement [4,6,7,9,10,11,12,13,14,15,16,17,18,19]. This finding highlights the overall effectiveness of ECT even when technical parameters were not clearly detailed and properly reported in the literature.

Regarding electrode placement, right unilateral ECT showed symptom reduction in 242 patients, while bilateral ECT resulted in improvement in 310 patients, with no reported non-responders in either group [7,10,13,20,21,22,23]. Similarly, bitemporal ECT demonstrated symptom reduction in 121 participants (100%) [9,21,24]. In contrast, unilateral ECT (non-specified laterality) presented a slightly lower response rate, with 96 patients (70.1%) showing symptom reduction and 41 patients (29.9%) reporting no improvement [15,25]. A comparable pattern was observed in bifrontotemporal ECT, where 83 participants (85.6%) improved, while 14 patients (14.4%) did not respond [16,26].

ECT administered with different pulse widths also showed favorable outcomes. Brief pulse ECT resulted in symptom reduction in 50 patients (100%), while ultra-brief pulse ECT demonstrated improvement in 22 participants (100%) [17,27,28]. Similarly, variations in stimulation intensity, including 600 milliampere (mA), 700 mA, and 800 mA protocols, were associated with symptom reduction in all reported cases, totaling 62 participants, with no documented non-responders [18,29]. Other specific configurations, such as bidirectional bitemporal ECT (150 patients), bilateral ECT administered three times per week (44 patients), and bifrontotemporal ECT once per week (20 patients), consistently demonstrated symptom reduction in 100% of treated participants [19,30,31,32]. Less frequently reported placements, including temporoparietal ECT, also showed symptom improvement, although these findings were based on a very limited number of cases [33].

Overall, across all ECT modalities analyzed, most patients exhibited a reduction in depressive symptoms, underscoring the robustness of ECT as an effective intervention in treatment-resistant depression [4,7,21].

#### 3.4.3. Transcranial Magnetic Stimulation (TMS) Alone

A total of 4862 participants received TMS alone. Symptom reduction was observed across several standard and non-standard rTMS protocols, although interpretation was limited by heterogeneity in stimulation target, frequency, laterality, coil type, session number, and outcome definitions [3,34,35,36,37,38,39,40,41]. TMS with unspecified parameters was applied in 1007 patients and showed symptom reduction in all cases, as well as repetitive stimulation without detailed frequency, which showed symptom reduction in all 860 patients [35,37]. In contrast, 701 participants who received sham therapy showed symptom mitigation, while 185 did not [36,42].

The largest sample corresponded to left dorsolateral prefrontal cortex stimulation (LDPFC), with a total of 1070 participants showing symptom reduction [3,38,43,44,45,46]. Additionally, repetitive 10 Hz stimulation was associated with symptom reduction in a substantial proportion of participants (n = 517), although non-response was also reported (n = 91) [39,47]. Several anatomical and technical variants of LDPFC and left prefrontal cortex stimulation (LPFC) also demonstrated symptom reduction such as LPFC stimulation (n = 168), dorsoprefrontal cortex stimulation (n = 200), left LDPFC stimulation on 5.5 cm anterior mid sagittal plane (n = 14), left DLPFC with TCD 12k (n = 15) and active stimulation over the anterior portion of the left middle frontal gyrus stimulation (n = 9) [40,48,49,50,51]. Symptom reduction was also observed with low-frequency right DLPFC stimulation in 184 participants, 1 Hz left DLPFC stimulation in 23 participants, low frequency left DLPFC stimulation in 10 participants, and low-frequency stimulation with unspecified laterality in 10 participants [41,52,53,54]. High-frequency stimulation also demonstrated clinical benefit with a lack of non-responders, including 20 Hz rTMS (n = 57), left DLPFC stimulation (n = 59), high-frequency left DLPFC stimulation (n = 59), and high-frequency stimulation with unspecified parameters (n = 21) [43,55,56,57]. Conversely, right DLPFC stimulation with unspecified frequency showed symptom reduction in 83 participants [58].

Bilateral approaches including bilateral dorsolateral and ventrolateral prefrontal cortex stimulation (n = 25), bilateral dorsolateral prefrontal cortex stimulation alone (n = 113), occipital repetitive stimulation (n = 24), six-point bilateral stimulation (n = 57), and bilateral stimulation not specified (n = 95) were associated with symptom reduction in all participants [44,59,60,61,62]. Sequential stimulation protocols such as synchronized stimulation, right-to-left DLPFC stimulation, and TMS followed by sham stimulation also demonstrated symptom reduction (n = 88, n = 74, and n = 20, respectively) [63,64,65].

In addition to monotherapy approaches, several studies reported outcomes for combined neuromodulation and pharmacological treatments, as well as mixed and control conditions. These modalities encompassed a smaller but clinically relevant proportion of the total sample and consistently demonstrated high rates of symptom reduction when active treatments were employed [2,74,75,76]. The only TMS modalities that did not show a response to treatment were low-frequency right sided stimulation and flipped-coil TMS [72,73].

#### 3.4.4. Electroconvulsive Therapy Combined with Pharmacotherapy

Among patients receiving ECT combined with pharmacological agents, a total of 350 participants were reported across different treatment protocols [77,78,79,80]. ECT combined with venlafaxine resulted in symptom reduction in 240 patients; similarly, bitemporal ECT combined with repeated ketamine administration (0.3 mg/kg) led to symptom improvement in 42 patients, while bitemporal ECT combined with ketamine 0.3 mg/kg administered once weekly resulted in symptom reduction in 46 patients [77,78,79]. Additionally, amitriptyline (150 mg) combined with ECT was associated with symptom improvement in 22 patients [80]. Across all ECT-pharmacotherapy combinations, no patients were reported as non-responders, indicating a 100% response rate within the included studies.

#### 3.4.5. Transcranial Magnetic Stimulation Combined with Pharmacotherapy

A total of 174 patients received TMS combined with antidepressant medication [81,82,83,84,85,86]. High-frequency rTMS targeting the left DLPFC using H1 or 8-coil systems combined with SSRIs or SNRIs resulted in symptom reduction in all treated patients [81,82,83,84]; other combined protocols also showed 100% response rates [85,86]. Furthermore, rTMS applied 5 cm anterior to the left DLPFC with concurrent antidepressant treatment and rTMS combined with pharmacotherapy without specified parameters, showed symptom reduction in 6 patients and 21 patients, respectively. Similarly to the combined ECT-pharmacotherapy group, no non-responders were reported in any of the included TMS–pharmacotherapy articles.

#### 3.4.6. Mixed Neuromodulation and Behavioral Interventions

Several studies reported outcomes from mixed or sequential treatment approaches, totaling 83 patients, all of whom experienced symptom reduction. These included unilateral ECT followed by high-frequency left prefrontal rTMS (20 Hz) in 12 patients, as well as ECT combined with right prefrontal 1 Hz rTMS, also involving 12 patients, both yielding a 100% response rate [87,88,89,90,91]. Other mixed interventions included sham treatment combined with active TMS (18 patients), ECT combined with sham rTMS (10 patients), transcranial direct current stimulation (tDCS) over the left DLPFC combined with cognitive-emotional training (20 patients), and low-frequency rTMS applied to the right prefrontal cortex combined with sleep deprivation (11 patients). All of these mixed treatment modalities reported symptom reduction in all treated participants [87,88,89,90,91]. Non-conventional stimulation techniques coupled with TMS also led to symptom control, including direct current stimulation at 2 mA (n = 14), high-dose direct current stimulation (n = 154), miniaturized stimulation (n = 17), and coil angled at 90 degrees from the scalp (n = 7) [66,67,68,69]. Heterogeneous protocols such as single biphasic DLPFC stimulation (n = 6) and high-theta/low-left DLPFC repetitive stimulation (n = 48) also showed symptom reduction [70,71].

#### 3.4.7. Reported Studies with Control Groups

Control and non-active treatment conditions demonstrated markedly different outcomes, as expected. In the placebo group, symptom improvement was reported in 29 patients (32.9%), while 59 patients (67.1%) did not show improvement. Similarly, patients receiving left and right-sided bilateral stimulation therapy (BLT) demonstrated symptom reduction in 34 patients (100% of those actually stimulated) [92,93,94]. In contrast, patients receiving muscle relaxants and anesthetics without ECT showed no symptom reduction, with 12 patients (100%) classified as non-responders. Likewise, in the no-treatment group, 289 patients (100%) failed to show symptom improvement, underscoring the clinical relevance of active neuromodulation and pharmacological interventions. Taken together, these findings demonstrate that active neuromodulation therapies, particularly ECT and TMS, were consistently associated with high rates of symptom reduction across a wide range of technical parameters, anatomical targets, and stimulation protocols [2,3,4,5,95]. Both ECT and TMS showed robust effectiveness when applied as monotherapies, while combined approaches involving pharmacotherapy or sequential neuromodulation were also associated with uniformly favorable outcomes in the analyzed samples. In contrast, control and non-active conditions, including placebo, absence of treatment, or anesthetic administration without ECT, were associated with substantially lower or absent rates of symptom improvement. Overall, the results underscore the broad applicability and clinical relevance of ECT and TMS-based interventions in patients with treatment-resistant depression, as reflected by consistent symptom reduction across diverse therapeutic configurations.

### 3.5. Non-Response to ECT and TMS

ECT or TMS represents a positive alternative for those with treatment-resistant depression [135,152]. Consequently, it is essential to compare outcomes across patients who are treatment-naïve, those with prior treatment failures, and individuals diagnosed with other depressive disorders, as these factors may substantially influence therapeutic response to ECT and TMS [12,161].

In our review, the number of patients who responded to treatment, defined by the presence of symptomatic improvement, varied according to the baseline depressive diagnosis, underscoring that treatment outcomes were influenced not only by the type of intervention (whether ECT, TMS, or combined therapy) but also by the underlying clinical characteristics associated with each depressive subtype [152,161,162]. For example, among patients diagnosed with major depressive disorder, a total of 7358 individuals were analyzed, of whom only 476 were classified as non-responders. In this group, 3443 patients responded to ECT, 3126 to TMS, and 313 to combined therapy (ECT + TMS), while no responders were identified among those receiving theta burst stimulation (TBS) [152,161]. Similarly, patients with treatment-resistant depression (n = 988) demonstrated a high overall response rate, with 98% of individuals showing clinical improvement. Specifically, 205 patients responded to ECT, 753 to TMS, and 11 to combined therapy, further supporting the effectiveness of neuromodulation strategies in this difficult-to-treat population [135,152].

The diagnostic subgroup with the highest proportion of non-responders was composed of individuals with unipolar or bipolar depression, in which 13.4% of patients (n = 137) failed to respond, all of whom received TMS. In contrast, within this group, 610 patients responded to ECT and 274 to TMS, highlighting differential treatment response across affective subtypes [161,162]. Moreover, six depressive subtypes demonstrated a 100% response rate across all reported treatments, encompassing diverse etiologies and sample sizes: vascular depression (106 patients), refractory depression (62 patients), postpartum depression (31 patients), late-life depression (141 patients), chronic stroke-associated depression (11 patients), and fibromyalgia-associated depression (13 patients) [152].

Patients not classified under any of the aforementioned categories were grouped as “other” depressive disorders. This group exhibited an overall response rate of 79.6%, with 54 patients responding to ECT, 32 to TMS, and 151 to combined therapy, while the remaining 20.4% (61 patients) were classified as non-responders, all of whom had been treated exclusively with TMS [152,161]. Notably, across all cases in which theta burst stimulation was applied (n = 360), no patients demonstrated symptomatic improvement within the analyzed samples [72,73]. However, theta-burst stimulation was reported separately from standard rTMS and sham/control conditions because of its distinct patterned stimulation structure. In the studies captured by our search and extraction framework, TBS was represented by a limited and heterogeneous subset of the literature [72,73]. Therefore, findings related to TBS should be interpreted descriptively and cautiously, and they should not be taken as evidence of a lack of therapeutic efficacy for TBS as a modality.

Overall, the therapies associated with the highest absolute number of responders were ECT and TMS, with 4600 and 4240 responders, respectively, reflecting their broader utilization across studies [152,161]. Although direct comparisons between therapies were challenging due to differences in sample size and diagnostic composition, 98.86% of patients receiving ECT alone and 94.39% of those receiving TMS alone were classified as responders, reinforcing the strong effectiveness of both neuromodulation techniques in depressive disorders [135,152,162].

### 3.6. Adverse Effects of ECT and TMS

Adverse-effect reporting was heterogeneous across the included studies. Of the 165 included articles, 44 explicitly reported adverse-effect data, whereas 121 did not provide detailed adverse-effect reporting. Therefore, the safety findings should be interpreted as descriptive counts of reported adverse-effect events among studies that assessed or reported them [21,57,90,110,114,121,124,148,155,156,176], rather than as incidence rates across the entire review population. Across studies with available adverse-effect data, 29 types of adverse effects were identified, including headache or local pain, confusion or delirium, memory impairment, cardiovascular complications, paresthesia or numbness, fatigue, and drowsiness. Because adverse-effect ascertainment, reporting thresholds, and follow-up duration varied substantially across studies, direct comparison of event rates between ECT and rTMS was not performed.

**Figure 2:**
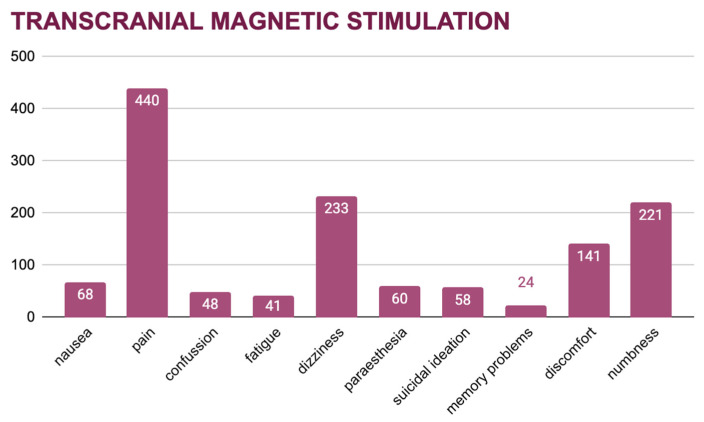
Absolute counts of reported adverse-effect events among studies reporting transcranial magnetic stimulation safety outcomes.

**Figure 3:**
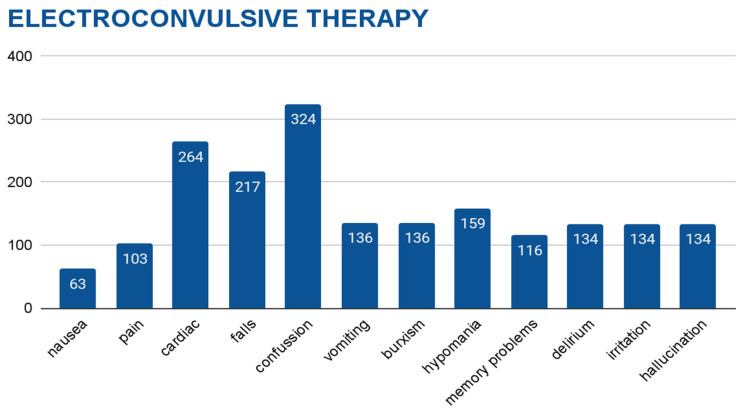
Absolute counts of reported adverse-effect events among studies reporting electroconvulsive therapy safety outcomes.

Among studies reporting adverse effects after rTMS, the most frequently described events were headache, scalp discomfort, stimulation-site pain, paresthesia, fatigue, and other mild transient symptoms. Among studies reporting adverse effects after ECT, the most frequently described events included confusion, memory impairment, delirium, cardiovascular events, and other postictal or anesthesia-related complications. These modality-specific patterns are clinically consistent with the procedural differences between rTMS and ECT; however, because most studies did not report adverse effects systematically, these findings should be interpreted as reported safety patterns rather than comparative adverse-event rates. The most predominant adverse effect was pain (headache), affecting 644 patients (out of these, 440 patients, representing 68,3%, underwent TMS alone) as shown in Figure 2 [21,57,90,110,114,121,124,148,155,156,176]. Confusion ranked second, with 372 patients affected, 324 (87,09%) of whom received ECT alone [84], as shown in Figure 3. The least reported adverse effect was drowsiness, with only 1 patient experiencing it [110], followed by fatigue, which was reported in 41 patients [124,156].

## 4. Discussion

Our scoping review aimed to determine the benefits and adverse effects of ECT, TMS, and their combined use in patients with major depressive disorder (MDD) and treatment-resistant depression (TRD). Our findings indicate that ECT provides the most consistent benefit in reducing depressive symptomatology, along with additional effects such as improvements in quality of life and increased brain-derived neurotrophic factor (BDNF) levels [2,4,135]. TMS also demonstrated significant clinical benefits, particularly in reducing baseline depression scores measured by standardized scales [6,12,153]. The sequential use of TMS and ECT showed benefits in symptom reduction and improvement in depression severity scores, although evidence remains limited [87,88,89,90,91]. Across all modalities, reductions in depression rating scales were observed, with comparable response rates between ECT and TMS [152,161,162]. Among studies that reported adverse-effect data, ECT was more commonly associated with cognitive, postictal, cardiovascular, and anesthesia-related events, whereas rTMS was more commonly associated with local and transient symptoms such as headache, scalp discomfort, paresthesia, fatigue, and stimulation-site pain. However, because adverse-effect reporting was absent or incomplete in many included studies, these findings should be interpreted as descriptive safety patterns rather than definitive comparative adverse-event rates.

In our review, ECT demonstrated the strongest evidence for clinical benefit, particularly in reducing depressive symptoms in patients with TRD. Across 55 articles evaluating ECT alone, 2781 patients experienced symptomatic improvement, alongside reported gains in quality of life, functional outcomes, and neurobiological markers such as increased BDNF levels [4,7,21]. These findings align with previous literature that consistently identifies ECT as one of the most effective interventions for severe and drug-resistant depression [135]. Similarly, TMS showed substantial benefits, with 3748 patients across 60 studies experiencing symptom improvement [3,34,35,36,37,38,39,40,41]. Although the magnitude of response was slightly lower than that observed with ECT, TMS demonstrated meaningful improvements in depressive symptoms and quality of life, supporting its role as an effective and less invasive alternative [103,165,174,175,176]. Prior systematic reviews have reported comparable antidepressant efficacy between TMS and ECT when evaluated using standardized symptom scales, particularly the HDRS [3,174,178,179,180]. The combined or sequential use of TMS and ECT was less frequently reported but suggested potential additive benefits, particularly in symptom reduction and baseline severity improvement [87,88,89,90,91]. While these findings are promising, the limited number of studies prevents definitive conclusions, highlighting the need for further controlled trials evaluating combined neuromodulation strategies.

Given the substantial heterogeneity observed across studies, it is clinically relevant to distinguish the stimulation parameters that currently have the strongest evidence from those that remain exploratory. For ECT, the most established clinical approaches were right unilateral, bitemporal, and bifrontal electrode placements, delivered using modern brief-pulse or ultrabrief-pulse devices. In the present review, right unilateral ECT, bilateral ECT, and bitemporal ECT were among the most frequently represented protocols and were consistently associated with symptomatic improvement. Bitemporal ECT has traditionally been favored when rapid clinical response is required, particularly in severe, psychotic, suicidal, or highly treatment-resistant depression, although it is also associated with a greater burden of cognitive adverse effects. Right unilateral ECT, particularly when delivered at adequately suprathreshold doses, is commonly used when preservation of cognitive function is a priority, while bifrontal ECT has been explored as an intermediate approach that may balance antidepressant efficacy and cognitive tolerability. Brief-pulse ECT remains a conventional standard, whereas ultrabrief-pulse right unilateral ECT is increasingly used to reduce cognitive adverse effects, although some evidence suggests that it may require higher stimulus dosing or a greater number of sessions to achieve comparable antidepressant efficacy [10,21,24,135]. On the other hand, for rTMS, the most established protocols are high-frequency stimulation of the left dorsolateral prefrontal cortex and low-frequency stimulation of the right dorsolateral prefrontal cortex. These approaches are supported by international evidence-based guidelines and have been repeatedly evaluated in randomized and naturalistic studies of major depressive disorder and treatment-resistant depression [5,22,40,42,62]. In practical clinical terms, high-frequency left DLPFC stimulation, most commonly around 10 Hz, is intended to increase cortical excitability in hypoactive left prefrontal networks, whereas low-frequency right DLPFC stimulation, commonly 1 Hz, is intended to reduce relative right prefrontal hyperexcitability. In the present review, left DLPFC stimulation represented the largest rTMS subgroup, with symptom reduction reported in 1070 participants, while 10 Hz stimulation was also associated with improvement in a substantial proportion of treated patients. Low-frequency right DLPFC stimulation was less frequently represented but also demonstrated clinical benefit in several studies.

By contrast, several approaches should be interpreted as experimental or emerging rather than standard clinical protocols. These include bilateral or sequential rTMS, accelerated rTMS schedules, theta-burst stimulation, deep-TMS using H-coils, MRI-guided or functional-connectivity-guided targeting, synchronized stimulation, occipital or multi-site protocols, and combined neuromodulation strategies involving ECT followed by rTMS or rTMS combined with cognitive-emotional training. Although many of these approaches showed promising outcomes in selected studies, they were usually represented by smaller samples, heterogeneous stimulation parameters, or limited replication. Therefore, their clinical role remains less clearly defined than conventional left DLPFC high-frequency rTMS, right DLPFC low-frequency rTMS, and standard ECT electrode configurations. This distinction is important because the high response rates observed across the overall dataset should not be interpreted as equivalent evidence for all protocols. Rather, the strongest clinical evidence currently supports standard ECT approaches, particularly bitemporal, bifrontal, and adequately dosed right unilateral ECT, and conventional rTMS protocols targeting the DLPFC, while newer individualized or accelerated protocols require further sham-controlled, adequately powered, and long-term comparative trials.

Reduction in standardized depression scales was a key outcome across all treatment modalities. Both ECT and rTMS were associated with clinically meaningful improvement across included studies. However, direct comparative interpretation is limited by heterogeneity in study populations, depression subtype, treatment-resistance definitions, stimulation protocols, outcome measures, follow-up duration, and clinical indication severity [152,161,162]. Importantly, ECT and rTMS are not necessarily used in exchangeable clinical populations. ECT is often considered in severe or highly refractory presentations, including psychotic depression, catatonia, severe suicidality, prior favorable response to ECT, or situations requiring rapid clinical improvement, whereas rTMS is generally positioned as a less invasive neuromodulatory option after inadequate response to pharmacological treatment [2,5,7]. Therefore, the descriptive outcome counts reported in this review should be interpreted as evidence-mapping summaries rather than as estimates of comparative or equivalent efficacy. These results are consistent with previous studies reporting similar effectiveness between both treatments despite differences in invasiveness and treatment protocols [152,161]. ECT showed sustained reductions across multiple scales, likely reflecting its long-standing clinical use and cumulative evidence base (134). TMS, while newer, has increasingly demonstrated robust reductions in HDRS, MADRS, and BDI scores in randomized, sham-controlled trials, reducing bias related to placebo effects [153]. Theta-burst stimulation was less consistently represented than standard rTMS protocols in the present review and was reported separately because of its distinct stimulation structure [72,73]. Therefore, the role of TBS in depressive disorders could not be evaluated definitively from our dataset. Rather than suggesting lack of efficacy, our findings indicate that TBS-specific conclusions are limited by the small number of captured studies, protocol heterogeneity, and the descriptive nature of this scoping review [72,73].

Clear differences emerged in the adverse effect profiles of ECT and TMS. In our review, ECT was associated with a broader and more severe range of side effects, particularly in older patients and those with cardiovascular comorbidities [114]. Reported complications included cardiac events, falls, cognitive disturbances, hallucinations, hypomania, bruxism, and irritability [114,135]. Neurological effects such as memory impairment, confusion, and delirium were more frequent following ECT, although most resolved within weeks after treatment. These findings are consistent with evidence indicating that seizure induction may disrupt memory consolidation in a therapy-dependent manner [114]. In contrast, TMS demonstrated a more favorable safety profile, with most adverse effects being mild, transient, and self-limited [10,11]. Common complaints included fatigue, scalp discomfort, paresthesia, and localized pain, likely related to stimulation of superficial nerves [10]. Although suicidality was reported in some cases, the literature suggests this was more closely related to underlying illness severity rather than the treatment itself [11]. Pain was a shared adverse effect between both therapies but was more prevalent in TMS, possibly due to stimulation of nociceptors in the scalp or trigeminal nerve branches [10]. Importantly, the overall tolerability of TMS may contribute to greater patient acceptance and adherence, particularly in populations at higher risk for ECT-related complications [165]. A more thorough comparison of the safety and possible adverse effects of using ECT versus rTMS can be found in Table 2.

Consistent with prior evidence, ECT remains one of the most effective treatments for severe and treatment-resistant depression, particularly in patients with repeated pharmacological failure [135]. However, its historical stigma and concerns regarding cognitive side effects continue to influence patient and clinician perceptions [12]. Our findings support previous observations that subjective improvement is often reported by both patients and clinicians, though methodological limitations, such as inadequate placebo comparisons, remain a concern in ECT research [159]. In contrast, TMS has emerged as a viable alternative that addresses many limitations associated with ECT, including invasiveness, cost, and adverse effect burden [165]. Its increasing use in recent years, supported by significant depressive symptom reduction [103], reflects growing clinical acceptance. While some studies suggest that ECT may offer more durable long-term benefits [164], the comparable short-term efficacy and superior safety profile of TMS make it particularly attractive in clinical practice.

Although the formal search for this scoping review ended in October 2022, several post-search publications provide important context for interpreting the present findings. These newer studies were not included in the formal synthesis, PRISMA flow diagram, or cumulative counts, but they highlight the rapid evolution of the field. For example, a recent Swedish register-based observational study comparing patients who had received both ECT and rTMS suggested greater antidepressant effectiveness for ECT in that clinical subgroup, reinforcing the need to avoid interpreting descriptive counts from heterogeneous studies as evidence of equivalence between modalities [181]. At the same time, updated guidelines and consensus literature continue to support the clinical role of non-invasive neuromodulation, including rTMS, within stepped and personalized treatment pathways for major depressive disorder [182]. Newer meta-analytic work also suggests that maintenance rTMS may help sustain remission or reduce relapse risk, although heterogeneity in maintenance schedules and patient selection remains substantial [183]. Similarly, recent evidence has refined the interpretation of theta-burst stimulation, showing that TBS should be considered a patterned rTMS approach with an emerging evidence base rather than an ineffective modality. Certainly, a network meta-analysis of 23 randomized controlled trials including 960 patients with depression found that several theta burst stimulation protocols were superior to sham, with cTBS over the right dorsolateral prefrontal cortex combined with iTBS over the left dorsolateral prefrontal cortex, and iTBS over the left dorsolateral prefrontal cortex, showing the most consistent benefits for response and depressive symptom improvement. Safety outcomes were comparable to sham, with no significant differences in all-cause discontinuation, switch to mania, or headache and treatment-site discomfort, supporting these protocols as having a favorable risk-benefit profile [184]. Finally, contemporary work on ECT continues to emphasize its strong antidepressant efficacy while underscoring the importance of cognitive monitoring, protocol individualization, and informed consent, particularly in patients at higher risk for autobiographical memory impairment or postictal cognitive effects [185]. These post-search publications do not change the descriptive nature of the present review, but they support our conclusion that future studies should prioritize standardized protocols, long-term follow-up, adverse-effect ascertainment, maintenance strategies, and clinically meaningful comparative designs.

In summary, both ECT and TMS demonstrate significant benefits in the treatment of TRD, with reductions in depressive symptoms and standardized rating scales [6,12,152,161]. ECT continues to show strong efficacy, particularly in severe cases, while TMS offers a safer and less invasive alternative with growing empirical support [135,165]. The combined use of both therapies represents a promising but still underexplored approach [87,88,89,90,91]. Future well-designed, placebo-controlled studies are essential to clarify long-term outcomes, optimize treatment sequencing, and guide individualized clinical decision-making.

### 4.1. Limitations

Although this scoping review provides a comprehensive overview of the available literature on ECT and TMS (165 articles encompassing 10,701 depression participants), various methodological limitations must be acknowledged. Although cumulative patient counts were reported to map the direction and frequency of outcomes, these figures are descriptive only and should not be interpreted as pooled response rates. The heterogeneity of study designs, stimulation parameters, outcome measures, follow-up periods, and response definitions precluded formal quantitative pooling and limits direct comparison between ECT, rTMS, TBS, and combined interventions. First, there was marked heterogeneity across included studies regarding stimulation parameters, including electrode placement, coil type, stimulation frequency, pulse width, intensity, number of sessions, and treatment duration; this variability limited the ability to draw structured comparisons between protocols and affected the stratification of outcomes according to technical parameters. The absence of standardized reporting in some studies further complicated the interpretation of treatment-specific effects and reduced reproducibility. Furthermore, the interpretation of theta-burst stimulation was limited by the small number of TBS-specific studies captured by the search strategy and by heterogeneity in stimulation protocols and reporting. Therefore, this review should not be interpreted as providing a definitive assessment of TBS efficacy or non-efficacy in depression. In addition, definitions of treatment-resistant depression were not uniform across the included studies. We accepted the terminology and operational definition used by each original study when clearly reported, rather than retrospectively applying a single definition of TRD across all articles. This may have introduced clinical heterogeneity, because some studies defined resistance according to failure of two adequate antidepressant trials, whereas others used broader terms such as refractory, drug-resistant, or medication-resistant depression. Therefore, diagnostic subgroup analyses should be interpreted descriptively rather than as strict comparisons between uniformly defined clinical populations. Second, the diagnostic composition was highly heterogeneous. Although most participants were diagnosed with major depressive disorder, an important proportion had treatment-resistant depression or other depressive subtypes with varying degrees of chronicity, severity, and prior treatment exposure; these clinical differences likely influenced response rates and may have inflated or attenuated perceived treatment efficacy. Furthermore, treatment-naïve individuals and patients with multiple pharmacological failures were frequently analyzed together, limiting the ability to determine differential responsiveness across illness trajectories. Third, many studies incorporated combined or adjunctive interventions, including concurrent pharmacotherapy, sham stimulation arms, or sequential neuromodulation approaches. In these cases, isolating the independent effect of active ECT or TMS was challenging, particularly in non-randomized designs. Although we excluded reviews and case reports, the inclusion of observational studies introduces inherent risks of cofounding, selection bias, performance bias, and publication bias. Fourth, adverse effects were inconsistently reported; a large proportion of included studies did not explicitly detail side effects, raising the possibility of underreporting. This limitation restricts the precision of safety comparisons between ECT and TMS and may underestimate the true incidence of cognitive or cardiovascular complications. Fifth, as a scoping review, the objective was to map and synthesize available evidence rather than perform quantitative pooling. Although risk of bias was assessed, no meta-analytic modeling was conducted, and therefore effect size magnitudes, heterogeneity statistics, and publication bias could not be formally evaluated. Therefore, the response rates reported should be interpreted descriptively rather than as definitive comparative efficacy estimates. An additional limitation is the search date. The literature search was completed up to October 2022, according to the original protocol, and was not updated before resubmission. Therefore, studies, guidelines, stimulation protocols, and practice recommendations published after this date were not captured. This limitation is particularly relevant in the rapidly evolving field of neuromodulation for depression, where newer evidence on accelerated rTMS, theta-burst stimulation, deep TMS, individualized targeting, maintenance strategies, and long-term safety may have emerged after the search window. In addition, the search was limited to PubMed-MEDLINE, Scopus, and the Virtual Health Library. Although these sources provide broad biomedical and multidisciplinary coverage, the absence of Embase and PsycINFO may have resulted in missed studies, especially from psychiatric and psychological intervention literature. For these reasons, the findings should be interpreted as a mapping of the evidence available within the prespecified search period rather than as an exhaustive synthesis of all evidence available at the time of publication. Finally, long-term outcomes were insufficiently represented in the literature; most studies focused on acute response, with limited follow-up data regarding relapse rates, maintenance strategies, durability of response, and long-term neurocognitive outcomes. These aspects are critical for informing clinical decision-making in treatment-resistant populations.

### 4.2. Conclusions

Our scoping review provides a comprehensive synthesis of the evidence available up to October 2022 regarding electroconvulsive therapy and repetitive transcranial magnetic stimulation in major depressive disorder and treatment-resistant depression. Across heterogeneous populations, study designs, and stimulation protocols, both interventions were associated with clinically meaningful reductions in depressive symptomatology. ECT remains a highly effective treatment option, particularly in severe, psychotic, suicidal, or highly refractory depressive episodes, and in clinical scenarios where rapid response is required [2,4,7]. rTMS, in contrast, represents a less invasive neuromodulatory approach with substantial evidence of antidepressant benefit and a generally favorable reported tolerability profile, particularly when delivered using established prefrontal stimulation protocols [3,5,6]. Because the included studies differed substantially in patient populations, clinical severity, treatment indication, stimulation parameters, outcome definitions, and follow-up duration, the present review does not establish comparative equivalence between ECT and rTMS. Treatment selection should therefore be guided by clinical urgency, depression severity, comorbidity burden, prior treatment history, safety considerations, treatment availability, and patient preference [2,5,7]. The heterogeneity identified across studies also underscores the need for clearer reporting of stimulation parameters, more consistent outcome definitions, systematic adverse-effect ascertainment, and longer-term follow-up. Future research should prioritize well-designed comparative studies, standardized protocol reporting, maintenance-treatment strategies, and clinically meaningful safety and functional outcomes to better define the role of ECT and rTMS within evidence-based depression care.

## Figures and Tables

**Table 1:** Number of articles per type of treatment reported in the literature.

Type of Treatment	Number of Articles
Electroconvulsive Therapy	55
Transcranial Magnetic Stimulation	95
Electroconvulsive Therapy and Transcranial Magnetic Stimulation	1
Electroconvulsive Therapy with Pharmacological Treatment	5
Transcranial Magnetic Stimulation with Pharmacological Treatment	8
Theta Burst Stimulation	1

**Table 2:** Comparative safety profile of ECT and rTMS in humans.

Domain	ECT	rTMS
Procedural requirements	Requires general anesthesia, neuromuscular blockade, seizure induction, and cardiorespiratory monitoring	Outpatient procedure, no anesthesia, no intentional seizure induction
Common adverse effects	Headache, nausea, myalgia, jaw pain, postictal confusion, disorientation, transient hypertension or tachycardia	Headache, scalp discomfort, stimulation-site pain, facial twitching, dizziness, fatigue, neck discomfort
Cognitive effects	Transient anterograde memory impairment, retrograde autobiographical memory complaints, postictal confusion, attention or executive disturbances	Usually cognitively neutral, clinically significant cognitive deterioration is uncommon
Cardiovascular effects	Transient autonomic changes, hypertension, tachycardia, bradycardia, arrhythmias, rare serious events	Usually minimal systemic cardiovascular effects, caution with implanted electronic devices
Serious but rare risks	Prolonged seizure, anesthetic complications, aspiration, dental or musculoskeletal injury, severe cardiovascular event, death	Seizure, treatment-emergent mania or hypomania, worsening depression or suicidality, hearing injury without protection
Higher-risk groups	Older adults, patients with cardiovascular disease, cerebrovascular disease, cognitive impairment, high anesthetic risk	Patients with epilepsy, structural brain lesions, recent stroke, medications lowering seizure threshold, metallic or electronic implants near the coil
Monitoring priorities	Pre-anesthetic assessment, ECG and vital signs, seizure duration, postictal recovery, cognitive monitoring	Screening for implants and seizure risk, motor threshold, stimulation parameters, hearing protection, mood and suicidality monitoring
Overall safety interpretation	Highly effective but associated with greater procedural and cognitive adverse-effect burden	Better tolerated and less invasive, with mostly mild and transient adverse effects

## Data Availability

The original contributions presented in this study are included in the article/Supplementary Material. Further inquiries can be directed to the corresponding author(s).

## Supplementary Materials

The following supporting information can be downloaded at https://www.mdpi.com/article/10.3390/jcm15135194/s1, Table S1: Search Strategy; Table S2: Bias Assessment. Table S3: Preferred Reporting Items for Systematic reviews and Meta-Analyses extension for Scoping Reviews (PRISMA-ScR) Checklist.
