# Supplementary material for: Electroconvulsive Therapy (ECT) and Repetitive Transcranial Magnetic Stimulation (rTMS), Benefits and Adverse Effects in Patients with Depression: A Scoping Review
Source: J Clin Med. 2026 Jul 2;15(13):5194. doi: 10.3390/jcm15135194 (PMC13363206; doi:10.3390/jcm15135194)
Supplement: Supplementary file 1 [file jcm-15-05194-s001.zip › jcm-4185987-supplementary.pdf]

Supplementary Materials: *"Electroconvulsive Therapy (ECT) and Repetitive Transcranial Magnetic Stimulation (rTMS), Benefits and Adverse Effects in Patients with Depression: A Scoping Review"*

Supplementary Table S1: Search Strategy

Database	Search Strategy
PubMed – MEDLINE	((Depression) OR (Depressive Disorders) OR (Depressive Syndrome) OR (Emotional Depression) OR (Chemical Depression) OR ("Depression"[Mesh])) AND ((Electroconvulsive Therap*) OR (Electroshock Therap*) OR (Electric Convulsive Therap*) OR (Electric Shock Therap*) OR ("Electroconvulsive Therapy"[Mesh]) OR (Transcranial Magnetic Stimulation) OR (Transcranial Magnetic Stimulation, Single Pulse) OR (Transcranial Magnetic Stimulation, Paired Pulse) OR (Transcranial Magnetic Stimulation, Repetitive) OR ("Transcranial Magnetic Stimulation"[Mesh])) AND ((Treatment Outcome) OR (Clinical Effectiveness) OR (Treatment Effectiveness) OR (Treatment Efficacy) OR (Clinical Efficacy) OR ("Treatment Outcome"[Mesh]))
Scopus	TITLE-ABS-KEY (((depression) OR (depressive AND disorders) OR (depressive AND syndrome) OR (emotional AND depression) OR (chemical AND depression)) AND ((electroconvulsive AND therapy) OR (electroshock AND therapy) OR (electric AND convulsive AND therapy) OR (electric AND shock AND therapy) OR (transcranial AND magnetic AND stimulation) OR (transcranial AND magnetic AND stimulation, AND single AND pulse) OR (transcranial AND magnetic AND stimulation, AND paired AND pulse) OR (transcranial AND magnetic AND stimulation, AND repetitive)) AND ((treatment AND outcome) OR (clinical AND effectiveness) OR (treatment AND effectiveness) OR (treatment AND efficacy) OR (clinical AND efficacy)))
Virtual Health Library (BVS)	((Depression) OR (Depressive AND Disorders) OR (Depressive AND Syndrome) OR (Emotional AND Depression) OR (Chemical AND Depression)) AND ((Electroconvulsive AND Therapy) OR (Electroshock AND Therapy) OR (Electric AND Convulsive AND Therapy) OR (Electric AND Shock AND Therapy) OR (Transcranial AND Magnetic AND Stimulation) OR (Transcranial AND Magnetic AND Stimulation, Single AND Pulse) OR (Transcranial AND Magnetic AND Stimulation, Paired AND Pulse) OR (Transcranial AND Magnetic AND Stimulation, Repetitive)) AND ((Treatment AND Outcome) OR (Clinical AND Effectiveness) OR (Treatment AND Effectiveness) OR (Treatment AND Efficacy) OR (Clinical AND Efficacy))

Supplementary Table S2: Bias Assessment

Nº	Author	DOI	Year	Country	% YES	Type of Bias
1	Mohamed A.Abdelnaim	10.3389/fpsyt.2019.00929	2020	Germany	71.4	MODERATE
2	Christopher C. Abbott	10.1016/j.jagp.2020.06.008	2020	USA	78.6	MODERATE

3	IAN M. ANDERSON	10.1192/bjp.bp.106.028019	2007	England	71.4	MODERATE
4	Andreou	10.1038/s41398-022-01960-8	2022	USA	57.0	MODERATE
5	M Argyelan	10.1038/tp.2016.54	2016	USA	57.0	MODERATE
6	Arici	10.1017/S1092852920001807	2020	Italy	92.9	MINIMUM
7	Azadeh Atashnama	10.1177/1039856220917070	2020	Australia	85.7	MINIMUM
8	William J. Burke	10.1111/j.1532-5415.1987.tb01397.x	1987	USA	85.7	MINIMUM
9	Jan Malte Bumb	10.1007/s00406-014-0543-3	2014	Germany	85.7	MINIMUM
10	Filip Bouckaert	10.1503/jpn.140322	2016	Belgium	71.4	MODERATE
11	P. R. Bosboom	10.1002/da.20144	2006	Netherlands	85.7	MINIMUM
12	Raphael Boechat-Barros	10.1590/s1516-44462004000200006	2004	Brasil	85.7	MINIMUM
13	Hal Blumenfeld	10.1016/S0925-4927(03)00073-8	2003	USA	77.7	MODERATE
14	Daniel M. Blumberger	10.1503/jpn.150265	2016	Canada	85.7	MINIMUM
15	Black	10.1016/0010-440X(87)90082-4	1987	USA	64.3	MODERATE
16	Tor Magne Bjølseth	10.1016/j.jad.2014.12.054	2015	Norway	92.9	MINIMUM
17	Bhatia	10.1097/00124509-199912000-00006	1999	USA	77.8	MODERATE
18	Siwei Baia	10.1016/j.eurpsy.2019.05.006	2019	Australia	57.1	MODERATE
19	Chris Baeken	10.1016/j.jad.2013.07.008	2013	Belgium	71.4	MODERATE
20	Chris Baeken	10.1016/j.clinph.2017.06.243	2017	Belgium	75.0	MODERATE
21	Christine A. Conelea	10.1016/j.jad.2017.03.063	2017	USA	85.7	MINIMUM
22	Carmen Concerto	10.3109/13651501.2015.1084329	2015	Italy	78.6	MODERATE
23	Eliza A. Coleman	10.1016/0006-3223(95)00185-9	1996	USA	50.0	MODERATE
24	Roni B. Cohen	10.1002/da.20486	2009	Brasil	78.6	MODERATE
25	Cristina Ciobanu	10.1016/j.jad.2013.03.024	2013	France	78.5	MODERATE
26	S. Cinar	10.1016/j.jad.2009.11.002	2009	Netherlands	85.7	MINIMUM
27	Andrei V. Chistyakova	10.1016/j.clinph.2004.09.008	2005	Ireland	71.4	MODERATE
28	Xing Chen	10.3389/fpsyt.2022.951595	2022	China	64.3	MODERATE
29	Shaw-Ji Chen	10.2147/NDT.S40466	2013	China	78.6	MODERATE
30	Cheng-Chung Chena	10.1097/YIC.0000000000000167	2017	Taiwan	78.6	MODERATE
31	Rogelio A. Cattan	10.1111/j.1532-5415.1990.tb01465.x	1990	USA	85.7	MINIMUM
32	Cash	10.1001/jamapsychiatry.2020.3794	2020	USA	42.5	HIGH

33	Silvia Casarotto	10.1007/s10548-012-0256-8	2012	Italy	75.0	MODERATE
34	Linda L. Carpenter	10.1002/da.21969	2012		85.7	MINIMUM
35	Carpenter	10.1016/j.brs.2017.06.003	2017	USA	85.7	MINIMUM
36	Marta Cano	10.1176/appi.neuropsych.18080177	2022	USA	75.0	MODERATE
37	Hannah Ford	10.1016/j.jad.2021.01.009.	2021	Australia	57.1	MODERATE
38	Corlier	10.1016/j.jad.2020.01.068	2020	USA	75.0	MODERATE
39	Corlier	10.1017/S0033291721002178	2021	England	71.4	MODERATE
40	Corlier	10.1093/cercor/bhz035	2019	Place: United States	75.0	MODERATE
41	Croarkin	10.1089/cap.2011.0054	2012		83.3	MINIMUM
42	Croarkin	10.1038/s41386-020-00829-y	2020	England	92.9	MINIMUM
43	Dalhuisen	10.1007/s00406-020-01135-w	2021	Germany	83.3	MINIMUM
44	Dong	10.1016/j.psychres.2019.112573	2019	Ireland	71.4	MODERATE
45	Dubin	10.1503/jpn.150223	2016	Canada	85.7	MINIMUM
46	Dumas	10.1186/1477-7525-10-87	2012		64.3	MODERATE
47	Durmaz	10.1080/24750573.2017.1293239	2017		85.7	MINIMUM
48	Eche	10.3389/fpsyt.2012.00013	2012	Switzerland	71.4	MODERATE
49	Ende	10.1001/archpsyc.57.10.937	2000	USA	66.7	MODERATE
50	Enneking	10.1016/j.brs.2020.03.018	2020	USA	58.3	MODERATE
51	Eshel	10.1038/s41386-020-0633-z	2020	England	85.7	MINIMUM
52	Fabre	10.1002/gps.1172	2004	England	71.4	MODERATE
53	Ferrucci	10.1016/j.jad.2009.02.015	2009		50.0	MODERATE
54	Filipčić	10.1016/j.jpsychires.2019.04.020	2019	England	92.9	MINIMUM
55	Fitzgerald	10.1038/npp.2008.233	2009	England	64.3	MODERATE
56	Fox	10.1097/00124509-200103000-00013	2001		77.8	MODERATE
57	Fukuda	10.1097/WNR.0000000000001523	2020		64.0	MODERATE
58	Furtado	10.1016/j.pscychresns.2012.02.004	2012	Ireland	71.0	MODERATE
59	Garcia	10.1016/j.brs.2009.06.001	2010	USA	78.6	MODERATE
60	García-Anaya	-	2011	Spain	71.4	MODERATE
61	García-Toro	10.1136/jnnp.71.4.546	2001	England	50.0	MODERATE

62	Ge	10.1016/j.pnpbp.2019.01.012	2019	USA	71.4	MODERATE
63	LauraGedge	10.3389/fpsyt.2012.00012	2012	Canada	78.6	MODERATE
64	Mark S. George	10.1176/ajp.154.12.1752	1997	USA	57.1	MODERATE
65	Andrew Giama	https://doi.org/10.1016/j.jadr.2021.100162	2021	Australia	71.4	MODERATE
66	Kate EM Godfrey	PMID: 31647794	2019	New Zealand	50.0	MODERATE
67	Michael C. Gold	https://doi.org/10.1016/j.brs.2022.01.007	2022	USA	66.7	MODERATE
68	Niall Gormley	10.1017/S0790966700004961	1999	England	71.4	MODERATE
69	Leon Grunhaus	10.1016/s0006-3223(99)00254-1	2000	Israel	57.1	MODERATE
70	Leon Grunhaus	10.1002/hup.418	2002	Israel	50.0	MODERATE
71	Gryglewski Gregor	10.1093/IJNP/PYZ059	2019	Austria	57.1	MODERATE
72	Gregor Gryglewski	10.1192/bjp.2018.224	2018	Austria	64.0	MODERATE
73	McLeod Frampton Gwynette	10.1002/aur.2266	2020	USA	64.0	MODERATE
74	Itay Hadas	10.1038/s41398-020-01042-7	2020	Canada	42.9	HIGH
75	Dakota Hadley	10.1097/YCT.0b013e3181ce1a8c	2011	USA	71.4	MODERATE
76	Poul Erik Buchholtz Hansen	10.1097/YCT.0b013e3181d77645	2011	Denmark	85.7	MINIMUM
77	POUL ERIK BUCHHOLTZ HANSEN	10.1080/08039480410011678	2004	Denmark	85.7	MINIMUM
78	Armand Hausmann	10.1097/YCT.0000000000000547	2019	Germany	71.0	MODERATE
79	Shunsuke Hayasaka	10.1111/pcn.12547	2017	Japan	64.0	MODERATE
80	HE Ming-li	10.3760/cma.j.issn.0366-6999.2011.12.004	2011	China	92.9	MINIMUM
81	Willemijn T.C.J. Heijnen⁎	10.1016/j.jpsychires.2018.11.014	2019	Netherlands	71.4	MODERATE
82	Tal Herbsman	10.1016/j.biopsych.2009.04.034	2009	USA	78.6	MODERATE
83	R. N. HERRINGTON A.	10.1016/S0140-6736(74)90936-2	1974	UK	50.0	MODERATE
84	Gokben Hizli Sayar	10.2147/NDT.S44241	2013	Turkey	78.0	MODERATE
85	Gökben Hızlı Sayar	10.1007/s00737-013-0397-0	2014	Turkey	71.0	MODERATE
86	Paul E. Holtzheimer	10.1002/da.20731	2010	USA	64.3	MODERATE
87	Brenton Hordacre	10.1007/s00415-020-10315-6	2020	Australia	92.0	MINIMUM
88	Kate E Hoy	10.1089/neu.2018.6097	2019	Australia	85.0	MINIMUM
89	Chih-Chia Huang	10.1016/S1726-4901(09)70209-6	2005	Taiwan	78.0	MODERATE
90	Martti J. Huuhka a	10.1016/j.ejpain.2003.11.001	2004	Finland	71.0	MODERATE

91	Martti Huuhka	10.1097/00124509-200409000-00010	2004	Finland	64.3	MODERATE
92	Haruki Ikawa	https://dx.doi.org/10.1002/npr2.12290	2022	Japan	78.6	MODERATE
93	Janakiramaiah N	10.1111/j.1600-0447.1998.tb10090.x	1998	India	92.9	MINIMUM
94	Philip G. Janicak	10.1017/S1092852913000357	2013	USA	92.9	MINIMUM
95	Venu Gopal Jhanwar	10.4103/0253-7176.85406	2011	India	64.3	MODERATE
96	Xue Jiang MM	10.1111/appy.12411	2020	China	78.6	MODERATE
97	Xi-long Jin	10.1097/NMD.0000000000000493	2016	China	85.0	MINIMUM
98	Yi Jin and Bill Phillips	10.1186/1471-244X-14-13	2014	USA	92.0	MINIMUM
99	Ricardo E. Jorge	10.1001/archgenpsychiatry.2007.45	2008	USA	78.6	MODERATE
100	Michel R. Ju	10.1016/j.jad.2005.09.005	2005	The Netherlands	78.0	MODERATE
101	Tyler S. Kaster	10.1038/s41386-018-0121-x	2018	Canada	92.9	MINIMUM
102	Mitra Keshtkar	10.1097/YCT.0b013e318221b31c	2011	Iran	78.6	MODERATE
103	Abrams	10.1001/archpsyc.1991.01810320070010	1991	USA	57.1	MODERATE
104	Kito	10.1111/j.1440-1819.2010.02183.x	2010	Japan	78.6	MODERATE
105	Klein	10.1001/archpsyc.56.4.315	2015	Israel	78.6	MODERATE
106	Krstić	10.1097/YCT.0000000000000099	2014	Serbia	78.6	MODERATE
107	Lee	https://doi.org/10.9758/cpn.2019.17.3.409	2019	Korea	78.6	MODERATE
108	Lerer	10.1176/ajp.152.4.564	1995	Israel	78.6	MODERATE
109	Leuchter	10.1016/j.brs.2015.05.005	2015	USA	78.6	MODERATE
110	Lin	10.1093/ijnp/pyx022	2017	Taiwan	85.0	MINIMUM
111	Ma	10.1002/brb3.1654	2020	China	78.6	MODERATE
112	MacMaster	10.3389/fpsyt.2019.00170	2019	Canada	64.3	MODERATE
113	Martínez-Amorós	10.1097/YCT.0b013e31823e2705	2012	Spain	78.6	MODERATE
114	Martin	10.1093/ijnp/pyv067	2015	Australia	78.6	MODERATE
115	May	10.1177/1039856219828134	2019	Australia	78.6	MODERATE
116	McCall	10.1192/bjp.185.5.405	2004	UK	78.6	MODERATE
117	McCall	10.1016/j.jpsychires.2017.11.001	2017	USA	71.4	MODERATE
118	McClintock	10.1002/da.22988	2019	USA	92.9	MINIMUM
119	McGirr	10.1016/j.jad.2014.10.068	2014	Canada	50.0	MODERATE
120	Mogg	10.1017/S0033291707001663	2007	UK	78.6	MODERATE

121	Moreno	10.1016/j.jad.2019.12.009	2019	Brazil	78.6	MODERATE
122	Moser	10.1212/WNL.58.8.1288	2002	USA	50.0	MODERATE
123	Ng	10.1097/00124509-200012000-00007	2000	Australia	50.0	MODERATE
124	Nikolin	10.1111/ejn.14656	2019	Australia	78.6	MODERATE
125	O'Connor	10.1097/00146965-200306000-00005	2002	USA	50.0	MODERATE
126	O'Leary	10.1002/gps.930090711	1994	UK	50.0	MODERATE
127	Obbels	10.1016/j.jagp.2019.04.006	2019	Belgium	78.6	MODERATE
128	Oliveira-Maia	10.1016/j.brs.2017.03.013	2017	USA	50.0	MODERATE
129	Ozcan	10.1016/j.psychres.2020.112873	2020	Turkey	91.6	MINIMUM
130	Pallanti	10.1159/000336999	2012	Italy	78.6	MODERATE
131	Park	10.1016/j.jpsychires.2020.04.012	2020	Korea	78.6	MODERATE
132	Akpinar	10.55730/1300-0144.5441	2022	Turkey	78.6	MODERATE
133	Pridmore Saxby	10.1002/1520-6394(2000)12:3<118::AID-DA2>3.0.CO;2-G	2000	Australia	71.4	MODERATE
134	Pridmore Saxby	10.1016/j.brs.2018.05.013	2018	Australia	66.6	MODERATE
135	Pridmore Saxby	https://doi.org/10.1177/1039856220943033	2020	Australia	58.3	MODERATE
136	Riddle William	10.1002/hup.470100306	1995	United Kingdom	71.4	MODERATE
137	Robin A	10.1192/bjp.141.4.357	1982	United Kingdom	50.0	MODERATE
138	Sanacora Gerard	10.1176/appi.ajp.160.3.577	2003	-	50.0	MODERATE
139	Sartorious A	DOI 10.1055/s-2006-950395	2006	Germany	28.6	HIGH
140	Schrijvers Didier	10.1159/000341881	2012	Belgium	78.6	MODERATE
141	Lea Sirignano	https://doi.org/10.1038/s41398-021-01474-9	2021	Germany	57.1	MODERATE
142	Song Ruize	https://doi.org/10.3389/fpsyt.2021.678384	2021	China	57.1	MODERATE
143	Sonmez A	DOI: https://doi.org/10.1016/j.pnpbp.2019.109763	2019	United States	78.6	MODERATE
144	William M. Stern	10.1176/jnp.2007.19.2.179	2007	United States	78.6	MODERATE
145	Hannah Stirton	https://doi.org/10.1371/journal.pone.0246592	2021	United States	75.0	MODERATE
146	Zvezdana Stojanovic	Doi: 10.3109/15622975.2016.1174299	2016	Serbia	78.6	MODERATE
147	Jie Tong	https:// doi.org/10.3390/brainsci11060765	2021	China	92.9	MINIMUM
148	Şakir Tosun	https://doi.org/10.1080/13651501.2019.1699118	2019	United States	75.0	MODERATE
149	KOUNOSUKE TSUCHIYAMA	10.1111/j.1440-1819.2005.01412.x	2005	Japan	66.6	MODERATE
150	Minna Valkonen-Korhonen	https://doi.org/10.1080/08039488.2018.1500640	2018	Finland	71.4	MODERATE

151	REINOUT VIEWEG	10.1080/09638239818003	1998	United Kingdom	64.3	MODERATE
152	D. N. VLISSIDES	doi:10.1192/bjp.141.3.239	1982	-	92.9	MINIMUM
153	Helena Voetterl	10.1016/j.jadr.2021.100250	2021	-	75.0	MODERATE
154	David H. Avery	doi:10.1016/j.biopsych.2005.07.003	2005	United States	92.8	MINIMUM
155	Anne Weigand	DOI: 10.1016/j.biopsych.2017.10.028	2017	-	71.4	MODERATE
156	Ronit Weizman	10.1016/0006-3223(95)00665-6	1996	Israel	66.6	MODERATE
157	Andrew Wilkinson	10.1002/gps.930080506	1993	United Kingdom	66.6	MODERATE
158	J Wittenborn	10.1097/00005053-196110000-00007	1961	United States	71.4	MODERATE
159	Yumeng Xin	https://doi.org/10.1016/j.brs.2021.12.008	2022	China	78.6	MODERATE
160	Lakshmi N. Yatham	doi: 10.1192/bjp.bp.109.069567	2010	-	75.0	MODERATE
161	Jerome A. Yesavage	doi:10.1001/jamapsychiatry.2018.1483	2018	-	78.6	MODERATE
162	Agustin G. Yip	10.1016/j.brs.2017.02.013	2017	-	57.1	MODERATE
163	Tingting Zhang	10.3389/fpsyt.2021.623765	2021	-	85.7	MINIMUM
164	Yao Zhang	10.3389/fpsyt.2022.859453	2022	China	75.0	MODERATE
165	Zhijun Zhang	https://doi.org/10.1007/s11427-020-1730-5	2020	China	85.7	MINIMUM

Supplementary Table S3: Preferred Reporting Items for Systematic reviews and Meta-Analyses extension for Scoping Reviews (PRISMA-ScR) Checklist

SECTION	ITEM	PRISMA-ScR CHECKLIST ITEM	REPORTED ON PAGE #
**TITLE**			
Title	1	Identify the report as a scoping review.	1
**ABSTRACT**			
Structured summary	2	Provide a structured summary that includes (as applicable): background, objectives, eligibility criteria, sources of evidence, charting methods, results, and conclusions that relate to the review questions and objectives.	1-2
**INTRODUCTION**			
Rationale	3	Describe the rationale for the review in the context of what is already known. Explain why the review questions/objectives lend themselves to a scoping review approach.	1-2
Objectives	4	Provide an explicit statement of the questions and objectives being addressed with reference to their key elements (e.g., population or participants, concepts, and context) or other relevant key elements used to conceptualize the review questions and/or objectives.	2-3
**METHODS**			

SECTION	ITEM	PRISMA-ScR CHECKLIST ITEM	REPORTED ON PAGE #
Protocol and registration	5	Indicate whether a review protocol exists; state if and where it can be accessed (e.g., a Web address); and if available, provide registration information, including the registration number.	3-4
Eligibility criteria	6	Specify characteristics of the sources of evidence used as eligibility criteria (e.g., years considered, language, and publication status), and provide a rationale.	3
Information sources*	7	Describe all information sources in the search (e.g., databases with dates of coverage and contact with authors to identify additional sources), as well as the date the most recent search was executed.	3
Search	8	Present the full electronic search strategy for at least 1 database, including any limits used, such that it could be repeated.	3
Selection of sources of evidence†	9	State the process for selecting sources of evidence (i.e., screening and eligibility) included in the scoping review.	3-5
Data charting process‡	10	Describe the methods of charting data from the included sources of evidence (e.g., calibrated forms or forms that have been tested by the team before their use, and whether data charting was done independently or in duplicate) and any processes for obtaining and confirming data from investigators.	3
Data items	11	List and define all variables for which data were sought and any assumptions and simplifications made.	3-4
Critical appraisal of individual sources of evidence§	12	If done, provide a rationale for conducting a critical appraisal of included sources of evidence; describe the methods used and how this information was used in any data synthesis (if appropriate).	3-4
Synthesis of results	13	Describe the methods of handling and summarizing the data that were charted.	3-4
**RESULTS**			
Selection of sources of evidence	14	Give numbers of sources of evidence screened, assessed for eligibility, and included in the review, with reasons for exclusions at each stage, ideally using a flow diagram.	4-5
Characteristics of sources of evidence	15	For each source of evidence, present characteristics for which data were charted and provide the citations.	3-4
Critical appraisal within sources of evidence	16	If done, present data on critical appraisal of included sources of evidence (see item 12).	3-4
Results of individual sources of evidence	17	For each included source of evidence, present the relevant data that were charted that relate to the review questions and objectives.	4-11
Synthesis of results	18	Summarize and/or present the charting results as they relate to the review questions and objectives.	5,7,11
**DISCUSSION**			
Summary of evidence	19	Summarize the main results (including an overview of concepts, themes, and types of evidence available), link to the review questions and objectives, and consider the relevance to key groups.	11-13

SECTION	ITEM	PRISMA-ScR CHECKLIST ITEM	REPORTED ON PAGE #
Limitations	20	Discuss the limitations of the scoping review process.	13-14
Conclusions	21	Provide a general interpretation of the results with respect to the review questions and objectives, as well as potential implications and/or next steps.	14
**FUNDING**			
Funding	22	Describe sources of funding for the included sources of evidence, as well as sources of funding for the scoping review. Describe the role of the funders of the scoping review.	15

JBI = Joanna Briggs Institute; PRISMA-ScR = Preferred Reporting Items for Systematic reviews and Meta-Analyses extension for Scoping Reviews.

* Where *sources of evidence* (see second footnote) are compiled from, such as bibliographic databases, social media platforms, and Web sites.

† A more inclusive/heterogeneous term used to account for the different types of evidence or data sources (e.g., quantitative and/or qualitative research, expert opinion, and policy documents) that may be eligible in a scoping review as opposed to only studies. This is not to be confused with *information sources* (see first footnote).

‡ The frameworks by Arksey and O'Malley (6) and Levac and colleagues (7) and the JBI guidance (4, 5) refer to the process of data extraction in a scoping review as data charting.

§ The process of systematically examining research evidence to assess its validity, results, and relevance before using it to inform a decision. This term is used for items 12 and 19 instead of "risk of bias" (which is more applicable to systematic reviews of interventions) to include and acknowledge the various sources of evidence that may be used in a scoping review (e.g., quantitative and/or qualitative research, expert opinion, and policy document).

From: Tricco AC, Lillie E, Zarin W, O'Brien KK, Colquhoun H, Levac D, et al. PRISMA Extension for Scoping Reviews (PRISMAScR): Checklist and Explanation. Ann Intern Med. 2018;169:467–473. doi: 10.7326/M18-0850.
